# Direct Intercellular Transport Mode of Filovirus Nucleocapsids

**DOI:** 10.3390/ijms26178485

**Published:** 2025-09-01

**Authors:** Catarina Harumi Oda Ibrahim, Yuki Takamatsu

**Affiliations:** 1 Department of Virology, Institute of Tropical Medicine, Nagasaki University, Nagasaki City 852-8523, Japan; ch-oda@nagasaki-u.ac.jp; 2 Graduate School of Tropical Medicine, Nagasaki University, Nagasaki City 852-8523, Japan

**Keywords:** Ebola virus, nucleocapsid, nucleocapsid-like structure, transport, live-cell imaging

## Abstract

Intercellular pathways of viral infection in host cells offer advantages, such as efficiency of viral spread and immune surveillance evasion, compared to cell-free viral infection. Therefore, some enveloped viruses present both cell-to-cell and cell-free forms of infection in the host organisms. In this study, we investigated the occurrence of Ebola virus (EBOV) and Marburg virus (MARV) nucleocapsid exchange in vitro between interconnected Huh7 cells using live-cell imaging methods. Moreover, through plasmid transfection methods, we demonstrated that nucleocapsid-like structures (NCLSs) formed with EBOV NP, VP35, VP24, and VP30 proteins can also be transported intercellularly to non-transfected cells through cell-to-cell contact regions in a process involving interaction with the host cell actin cytoskeleton. Our results provide further evidence of cell-to-cell transport as a mechanism of filovirus spread and support the need for further research in this field to develop new intervention methods targeting this transmission pathway.

## 1. Introduction

Ebola virus (EBOV) and Marburg virus (MARV) are members of the *family Filoviridae*, and include the *genera Orthoebolavirus*, *Cuevavirus*, *Orthomarburgvirus*, *Dianlovirus*, *Oblavirus*, *Striavirus*, *Tapjovirus*, and *Thamnovirus* [1]. Filoviruses are enveloped, non-segmented, negative-strand RNA viruses with filamentous shape [2,3,4]. Both diseases are characterized by severe hemorrhagic fever, leading to an almost 90% fatality rate [2,5,6]. Therefore, a better understanding of viral replication mechanisms may help in the development of antiviral drugs or therapies that target specific proteins or steps involved in this process to control viral spread [3].

**Figure 1:**
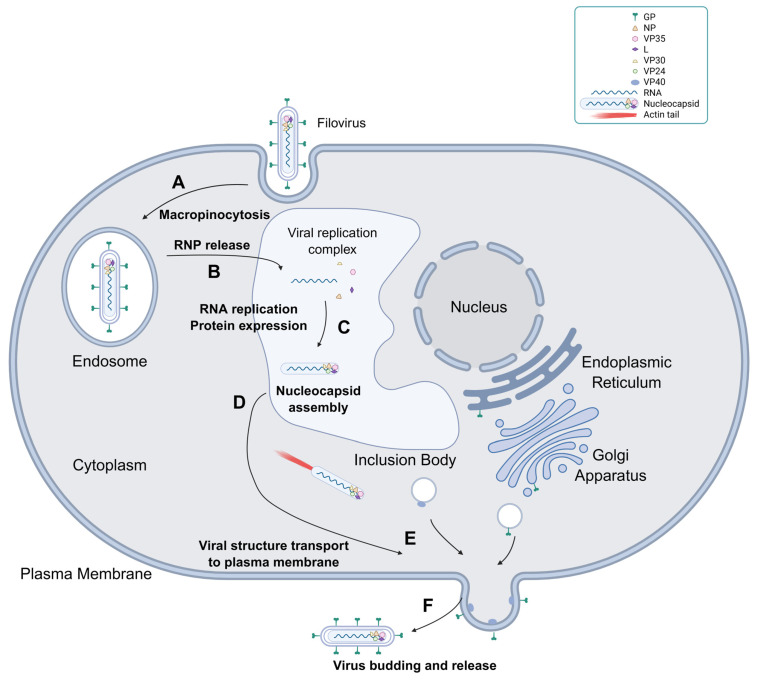
Scheme of the filovirus infection cycle. (**A**) Viral entry into host cells via macropinocytosis. (**B**) RNP release from the late endosomes into the cytoplasm. (**C**) Viral RNA replication and protein expression mediated by the viral replication complex (NP, VP35, L, and VP30) inside inclusion bodies. GP undergoes post-translational processing in the endoplasmic reticulum of the host cells. (**D**) Nucleocapsid assembly mediated by VP24. (**E**) The nucleocapsid is transported to the plasma membrane by the actin tail, whereas VP40 and GP are transported to the membrane by vesicles and begin to form filopodia. (**F**) Viral budding and release into the extracellular space via filopodia. The figure was created using BioRender (web version) https://BioRender.com/41hvjlo (accessed on 31 July 2025).

Viral entry into host cells is mediated by the interaction between EBOV glycoproteins (GPs) and the C-type lectin and phosphatidylserine receptors located on the host cell surface, allowing internalization of the viral particles by macropinocytosis (Figure 1A) [7,8,9]. After the late endosomal stages, the receptor-binding domain of GPs is exposed and interacts with the intracellular receptor Niemann–Pick C1 (NPC1), which is located in the endosomes, mediating viral ribonucleoprotein (RNP) release into the cytoplasm, beginning the viral replication process (Figure 1B) [2,10,11]. The viral replication complex, formed by nucleoprotein (NP), polymerase cofactor (VP35), L polymerase (L), and transcriptional activator (VP30), mediates RNA replication and protein expression, usually in the perinuclear regions of the host cell, with the formation of inclusion bodies, which are membraneless phase-separated structures with concentrated viral proteins that act as viral factories (Figure 1C) [10,12,13]. Afterwards, viral protein 24 (VP24) assembles the expressed viral components in nucleocapsid structures (Figure 1D), which are transported towards the plasma membrane, where matrix protein (VP40) and GPs are located, mainly towards filopodia structures enriched with VP40 (Figure 1E) [10,14]. Nucleocapsid movement in the cytoplasm depends on the interaction of the viral proteins NP, VP35, and VP24 with the actin filaments of the host cell [15]. These three viral proteins and host cell Arp2/3 complex signaling induce the formation of actin tails that direct nucleocapsid transport towards the cell periphery [16]. While GP is processed and transported to the plasma membrane via a secretion pathway involving the endoplasmic reticulum and Golgi apparatus [7,17], VP40 is transported by late endosomes (Figure 1E) [7,18]. VP40 promotes virus budding using endosomal complexes required for the transport (ESCRT) pathway to form filopodia, in which most viral budding and release to the extracellular space occurs, and a part of the host cell’s plasma membrane is used to form the viral envelope (Figure 1F) [6,10,14].

Interestingly, some studies have shown that EBOV may infect naïve cells through direct contact between neighboring cells, without the need for the formation of a cell-free form [19,20,21,22]. One study has suggested that GPs are involved in cell membrane adherence by forming viral synapses or promoting cell fusion, allowing the passage of nucleocapsids from infected cells to non-infected cells [19,21,22]. Another study suggested that viral infection induces the formation of intercellular tunneling nanotube (TNTs)—actin bridges that directly connect the cell cytoplasm for organelles, proteins, and signal exchange—in macrophages, the primary targets for infection, which allows the quick passage of nucleocapsids from infected cells to non-infected cells [20]. Considering that the intercellular spread of the virus has some advantages with regard to viral evasion from antibodies and the efficiency of viral particle transfer to other cells, as there is no need to go through the steps of viral encapsulation or budding for release, this form of transmission may help in viral spread at high titers, contributing to the severity and lethality of the disease [23,24,25].

In this study, we provide further visual evidence using live-cell imaging [26,27] under in vitro conditions, of EBOV (EBOV Zaire Mayinga strain) and MARV (MARV Musoke strain) nucleoprotein intercellular transport, both by actin-derived cellular extensions connecting neighboring cells and by neighboring cell adhesion. Furthermore, using the viral protein-expressing plasmid technique, we demonstrated that the same viral proteins involved in the interaction with actin molecules from host cells for intracellular transport of nucleocapsids [15] are also involved in intercellular transport. These results suggest the necessity of conducting further research on cell-to-cell transmission to develop treatments targeting this infection pathway.

## 2. Results

### 2.1. EBOV Intercellular Transport by Cellular Extension Bridges

**Figure 2:**
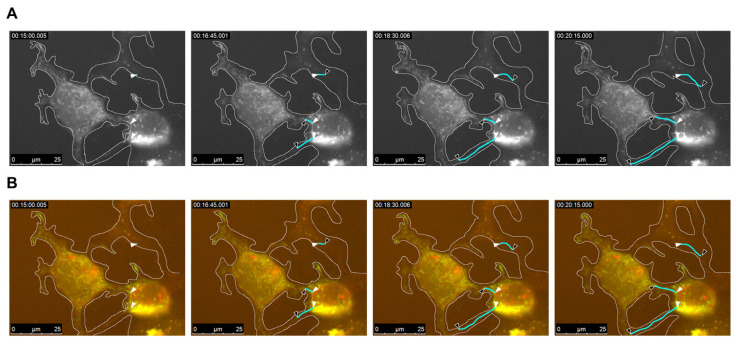
EBOV intercellular transport through nanotube structures. Huh7 cells were infected with EBOV and transfected with VP30-GFP and VP24-TagRFP markers at 1 h post-infection. Live-cell imaging was performed from 20 h post-infection for 30 min with a 3 s interval, using a 63× oil objective lens (see Video S1). Four time-lapse moments are presented in (**A**) grayscale and (**B**) color. Cell boundaries are marked in white. Cyan lines represent the trajectory of individual nucleocapsids, with white arrowhead as the origin point and black arrowhead as the finish point in each time frame. The time frames displayed in hours, minutes, and seconds are shown in the top left corner. Trajectory marking was performed using the ImageJ 1.54g software.

To determine whether EBOV infection induces cell-to-cell viral spread, Huh-7 cells were infected with live EBOV and, after 1 h, transfected with pCAGGS-VP30-GFP and VP24-TagRFP plasmids. Viral movement was recorded by live-cell time-lapse experiments after 24 h, using a Leica DMI6000B microscope (Germany) with a 63× oil objective lens inside BSL4, using VP30-GFP and VP24-TagRFP as trackers for nucleocapsid structures. The Leica Application Suite X 3.10.1 software was used for image stacking in video format (Video S1). Figure 2 shows the trajectories of three marked nucleocapsids between neighboring cells in grayscale (Figure 2A) and in color (Figure 2B). Although VP30 appeared to colocalize with nucleocapsids, not all VP24 was colocalized, as part of it was concentrated in round structures and the others appeared diffused in the cytoplasm (Figure 2B). This may be related to the difficulty of RFP-bound VP24 interaction with the virus. Regarding nucleocapsid movements, viruses circulate both toward and around the plasma membrane in the cytoplasm of host cells and across other cells, which are connected by fused membranes that are morphologically similar to TNTs (Figure 2). Nucleocapsids appeared to move freely in a bidirectional manner between connected cells through these TNT-like structures, suggesting that molecular exchange between these cells was not restricted, with cytoplasmic content shared by both cells through these tunnels (Video S1). This suggests that intercellular exchange of EBOV nucleocapsids is not only possible through these bridges connecting neighboring cells but is also a fast and efficient way of spreading a large quantity of viral particles to other cells without requiring the cell-free enveloped form of the virus.

### 2.2. MARV Intercellular Transport Through Neighboring Cell Contact

**Figure 3:**
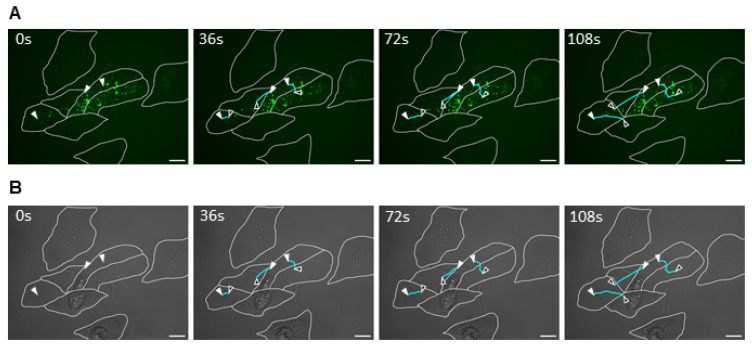
MARV intercellular exchange by cell-to-cell membrane contact. Huh7 cells were infected with MARV and transfected with VP30-GFP marker 1 h post-infection. After 40 h, live-cell imaging was performed with capture for 6 min at 6 s intervals, using a 63× oil immersed objective lens (see Video S2). (**A**) The GFP signal from VP30-GFP showing MARV nucleocapsid movement. (**B**) Transmitted light view of the same frame. The nucleocapsid trajectory is marked with white and black arrowheads and cyan lines, respectively. Trajectory marking was performed using the ImageJ 1.54g software. Scale bar: 25 μm.

Having observed the intercellular transport of EBOV viral nucleocapsids between infected cells through filopodia, the possibility of this phenomenon being observed in other filoviruses should also be considered. To test this, Marburg virus (MARV) was used for cell infection, considering its similarity to EBOV in terms of structure, genome, and nucleocapsid intracellular transport mechanism [4,6]. Similar to the EBOV experiment, Huh-7 cells were initially infected with MARV. After 1 h, cells were transfected with mVP30-GFP and incubated for 40 h. Using a Leica DMI6000B microscope with a 63× oil objective lens, live-cell time-lapse pictures were taken inside the BSL4 and analyzed using the Leica Application Suite X 3.10.0 software for video processing (Video S2). While nucleocapsid movement was observed using VP30-GFP signal (Figure 3A), the cell membrane limits were delimited by observing the transmitted light view of the infected cells (Figure 3B). For MARV infection, intercellular nucleocapsid exchange was observed; however, in contrast to EBOV infection, it was performed by direct adherence of neighboring cell membranes (Figure 3A). Although the membranes of these cells appeared to be intact, it is possible that the passage of nucleocapsids between them was facilitated by the formation of virological synapses or short tubular passages (Figure 3B). Whether the cellular interactions leading to intercellular exchange of viral nucleocapsids are different between EBOV and MARV cannot be confirmed in this experiment, considering the possibility that both viruses can perform TNT-like structures or viral synapse-dependent viral exchange, depending on intrinsic and extrinsic conditions. However, these experiments showed that at least in vitro, cell-to-cell viral transmission may occur for filoviruses, and it is possible that a conserved mechanism of these viruses is involved in this process.

### 2.3. EBOV NCLS Interaction with Actin Is Involved in Intercellular Viral Transmission

**Figure 4:**
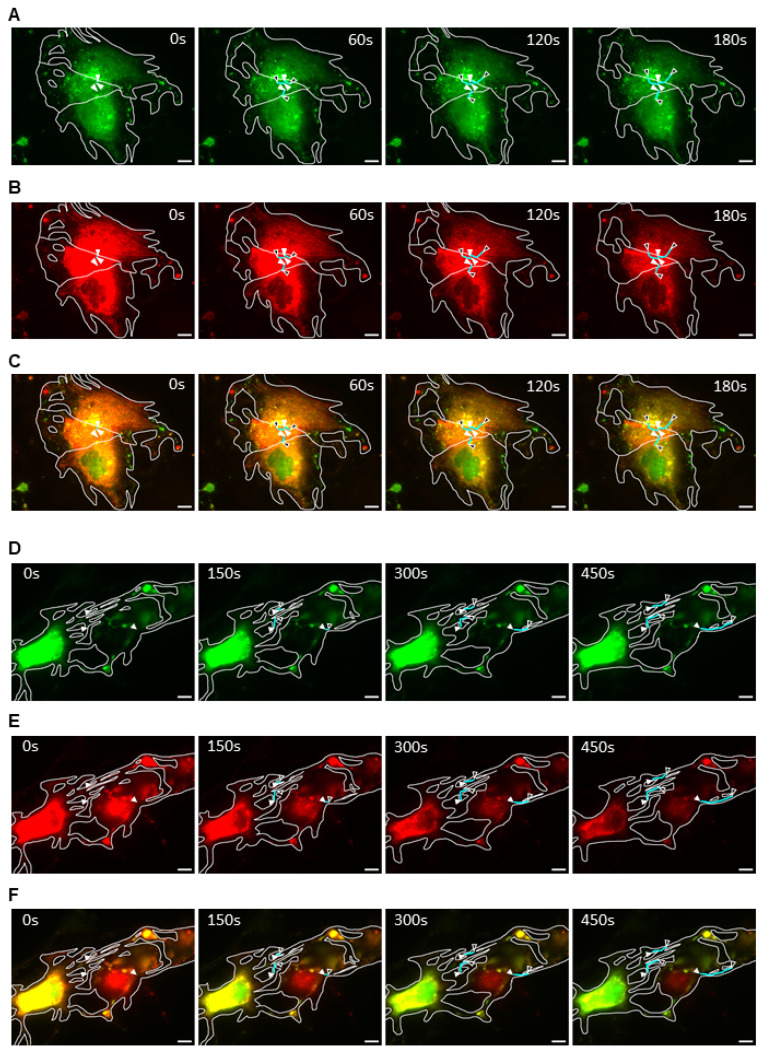
EBOV NCLS intercellular exchange between fused cells and nanotubes. Huh7 cells were transfected with EBOV NCLS components (NP, VP35, and VP24) and VP30-GFP tracker, together with TagRFP-actin. Twenty hours post-transfection, live-cell imaging was performed for 7.5 min at 6 s intervals using a 100× oil immersed objective lens (see Video S3). (**A**–**C**) NCLS movement between fused cells and (**D**–**F**) NCLS movement between cells connected with nanotubes, with (**A**,**D**) VP30-GFP, (**B**,**E**) TagRFP-actin, and (**C**,**F**) merged time-lapse views. A color balance saturation increase of 35% was applied to the RFP signal using ImageJ in (**B**,**C**) to allow its visualization. NCLS movement is represented by white and black arrowheads and cyan lines, respectively. Trajectory marking was performed using the ImageJ 1.54g software. Scale bar: 10 μm.

A previous study showed that NP, VP35, and VP24 proteins are the minimal components required for nucleocapsid transport by host cell actin in infected cells. Moreover, the expression of these proteins was sufficient to transport the NCLS to the plasma membrane during the budding stage [15]. To evaluate whether these components are also involved in the observed intercellular exchange of nucleocapsids, Huh7 cells were transfected with EBOV NP, VP35, VP24, VP30-GFP (for tracking), and an actin visualization. After 20 h, live-cell time-lapse pictures were taken using a Keyence All-in-One Fluorescence microscope BZ-X800(Tokyo, Japan), using a 100× oil objective, and the results were analyzed using Keyence BZ-X series 1.3 software for video processing (Video S3). These results showed that both NCLS and actin were colocalized in host cells and throughout the cytoplasm (Figure 4C,F). Additionally, intercellular transport was observed in both cell groups. The first group (Figure 4A–C, Video S3C) showed cell-to-cell NCLS transfer between fused cells, in which the membrane boundaries separating these cells were no longer visible, allowing free exchange of these viral structures. In comparison, the second group (Figure 4D–F, Video S3F) presented cells connected by TNT-like structures, allowing the transfer of NCLS through them. These results suggest that NCLS alone, which likely interacts with actin filaments, can promote the intercellular exchange of viral components without the need for membrane-associated viral proteins. In addition, intercellular connection forms vary between TNT-like structure formation and cell fusion, indicating that there may be multiple forms of cell interactions induced by EBOV proteins or cellular machinery.

### 2.4. EBOV NCLS Can Be Transferred to Naïve Cells by Cell-to-Cell Interactions

**Figure 5:**
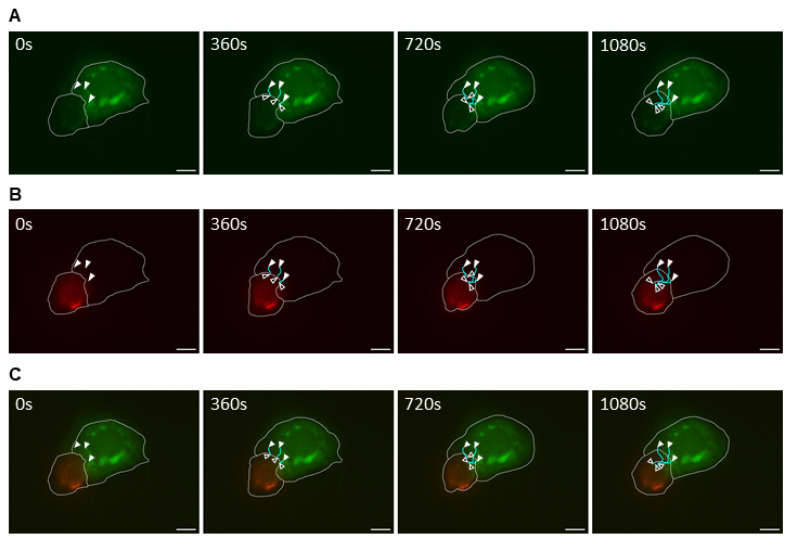
EBOV NCLS transport to naïve neighbor cells via cell-to-cell connections. Huh7 cells were divided into two groups: one with the transfection of NCLS components and VP30-GFP (infected model group) and the other with the actin marker (naïve model group). The NCLS-transfected cell group was cocultured with the naïve group at 22 h post-transfection and, an additional 20 h later, live-cell imaging was performed for 18 min at 6 s intervals, using a 63× oil immersed objective lens (Video S4). (**A**) NCLS-transfected cells, (**B**) TagRFP-actin-transfected cells, and (**C**) merged time-lapse view of interactions between cells from both groups. The NCLS trajectory is marked with white and black arrowheads, and cyan lines. Trajectory marking was performed using the ImageJ 1.54g software. Scale bar: 10 μm.

In all previous experiments, intercellular transport between the infected cells was observed. To determine whether this form of viral spread was possible in non-infected cells, Huh-7 cells were divided into two groups. In the first stage, EBOV NCLS protein and VP30-GFP were transfected into cells in one well, and TagRFP-actin was transfected into cells in another well. In the second stage, the cells were detached and the two cell populations were thoroughly mixed and seeded. Intercellular interactions were observed using live-cell imaging with a Nikon ECLIPSE TE2000-E microscope (Germany)(63× oil-immersion lens). The results were analyzed using Nikon NIS Elements 4.1 software and overlaid in video format (Video S4). Figure 5 shows the interactions between NCLS-expressing (Figure 5A) and TagRFP-actin-expressing cells (Figure 5B). In this case, although the cells did not appear fused, movement via viral synapses or small tunnels between the cells was possible (Figure 5C). This suggests that the NCLS is important for the intracellular movement of nucleocapsids between infected and uninfected cells.

## 3. Discussion

Cell-to-cell transmission of viruses, in addition to cell-free forms of infection, may be advantageous for viral transmission. While the cell-free form of the virus can be favorable for infection spread to distant host tissues and transmission to other organisms, cell-to-cell infection allows the direct transfer of viral particles to neighboring cells and the transfer of viral particles to cells that are not susceptible to the cell-free form of viral infection, as long as there is a connection between this cell and an infected cell, and the possibility of evasion from the host immune system, mainly against antibodies that target the cell-free form of the virus [23,28,29,30].

The current study demonstrates that intercellular exchange of viral nucleocapsids, occurring via cell fusion or intercellular bridges, may serve as a pathway for EBOV and MARV dissemination. This indicates that the conserved protein structures or interaction mechanisms shared by these viruses may be involved in this process. Further experiments are required to determine whether these connections function as virological synapses or tunneling nanotubes (TNTs), and to identify the cellular or viral components that mediate their formation. Future validation using electron microscopy techniques is essential to elucidate these mechanisms.

Moreover, we have shown that, similar to nucleocapsid intracellular transport, EBOV intercellular transport is possible with the viral proteins NP, VP35, and VP24 complex, the minimal components required for long-distance nucleocapsid transport by interacting with actin filaments of the host cell [15,16], including infection of naïve cells. Interestingly, NCLS intercellular transport was slower in comparison to previous studies regarding intracellular transport [15], particularly when they crossed narrow pathways (Figure 4D–F) or at the physical barriers between separate cells (Figure 4A–C and Figure 5), in which NCLS are moving shorter distances at a specific time frame, in comparison to when they are moving intracellularly, which suggests the presence of a certain resistance to intercellular NCLS movement. Although it is not possible to confirm that the same mechanisms observed for NCLS intracellular transport are also responsible for the intercellular transport, this finding may help guide other studies concerning NCLS affecting intercellular filoviral infection.

Furthermore, we demonstrated that under in vitro conditions, it is possible to observe intercellular filoviral nucleocapsid exchange between human hepatocyte-derived cells (Huh7). Considering that the liver is a key target organ during filoviral infection, future studies focusing on observing such infection patterns in vivo are highly valuable [2,5]. It is also plausible that the preference for intracellular versus intercellular transmission pathways varies depending on the cell type and extracellular environment. For example, Djurkovic et al. [20]. reported intercellular transmission of EBOV in macrophages and endothelial cells. Understanding how different cell types influence the mode of viral transmission could provide deeper insights into filoviral pathogenesis and facilitate the development of targeted therapeutic strategies.

However, our study had certain limitations. First, the mechanisms underlying the interaction of host cells during infection, which allows the transfer of viral nucleocapsids, remain mostly unknown. Previous studies have proposed that cleaved viral GPs polarized on the cell surface are involved in the formation of viral synapses with neighboring cells or induce cell fusion, or that VP40 interacting with the host cell’s plasma membrane may be involved in the elongation of the filopodia structure formed during viral budding towards other cells, forming TNT-like connections between them [19,20,21,22,26]. Our results suggest that NCLS are involved, considering that cell-to-cell NCLS transfer was observed in their presence alone. However, because it is not possible to confirm whether intercellular connections are formed by their influence or by the stress applied to cells during the transfection process, further studies on the effects of NCLS on host cell plasma membranes should be conducted. Another limitation of our study is that in each experiment, intercellular transport of the viral nucleocapsid or NCLS was observed in only a limited number of cells, making quantification challenging. Due to biases introduced during the selection of observation cells, it is difficult to specify the precise frequency of intercellular transport numerically. Intracellular transport was observed in approximately half of the cells under our experimental conditions, and intercellular transport was observed in approximately one-tenth of the cells. It remains unclear whether intercellular transmission among filoviruses is inherently rare, whether the culture conditions are suboptimal for detecting this process, whether this phenomenon occurs more frequently in other cell lines, or whether transfection procedures influence the results. Nevertheless, consistent observation of intercellular transport across multiple experiments suggests that the possibility of intercellular infection by filoviruses cannot be dismissed. Further detailed characterization of this phenomenon is required in future studies.

Research on other enveloped RNA viruses possessing both cell-to-cell and cell-free spread mechanisms may offer clues regarding the intercellular transmission mechanisms of filoviruses. Studies of severe acute respiratory syndrome coronavirus 2 (SARS-CoV-2) and human immunodeficiency virus 1 (HIV-1) have shown that their glycoproteins, which are involved in host–cell interactions and internalization of cell-free forms of the virus, can also promote intercellular transmission by inducing cell fusion and forming syncytia by influencing cytoskeletal rearrangement pathways [24,25,30,31]. Moreover, other studies on SARS-CoV-2, Chikungunya virus (CHIKV), and HIV-1 showed the induction of TNT formation during infection by interacting with proteins involved in controlling protrusion formation pathways and cytoskeleton polymerization or crossing over already-made TNTs, allowing the quick passage of viral particles without requiring a cell-free form [24,32,33,34,35]. Finally, studies on human T-lymphotropic virus-1 (HTLV-1), dengue virus (DENV), and HIV-1 have shown that the formation of viral synapses—stable cell junctions formed in regions polarized with host cell cytoskeleton proteins, viral envelope glycoproteins, and other viral components—allows the passage of virions, viral proteins, and viral RNA through the synaptic space with little damage to host cell membranes, considering that their membranes are not fused to each other [36,37,38,39,40,41].

Studies on the cell-to-cell spread of filoviruses are limited, and their mechanisms are still poorly understood. Our study presents visual evidence in vitro of EBOV and MARV nucleocapsid exchange between host cells through intercellular connections and that these nucleocapsids interact with host cell actin for this sort of transport. Considering the immunological response evasion and efficiency of viral particle spread advantages of intercellular viral transmission, further research focusing on the mechanisms of this form of spread and the regulation of cell-free and cell-to-cell transport should be conducted to develop treatments targeting this infection to limit viral spread in patients.

## 4. Materials and Methods

### 4.1. Cells and Viruses

Huh-7 (human hepatoma) cells were maintained in Dulbecco′s modified Eagle’s medium (DMEM; Wako, Tokyo, Japan) supplemented with 10% (*v*/*v*) fetal calf serum (FCS), 5 mM L-glutamine, 50 U/mL penicillin, and 50 µg/mL streptomycin (PS) at 37 °C and 5% CO_2_. These cells, in particular, were chosen because of their susceptibility to EBOV and MARV infection, transfection efficiency, and the convenience of viral nucleocapsid visualization by live-cell imaging owing to the large size of the cell cytoplasm. Given that the liver is a major target organ for filoviral infection, Huh-7 cells serve as an appropriate hepatic cell model for studying the mechanisms of viral dissemination. Live virus experiments were conducted using recombinant EBOV Zaire (Mayinga strain; GenBank accession number AF272001) and recombinant MARV (Musoke strain; GenBank accession DQ217792.1) were used in this study [27,42]. The use of recombinant viruses offers the advantage of obtaining the original genomic sequence of viral strain via reverse genetics, allowing experiments to be conducted with the virus to maintain its sequence fidelity and reduce the risk of mutations that can occur with multiple passages. Experiments involving infectious EBOV and MARV strains were performed at a biosafety level 4 (BSL-4) facility at the University of Marburg.

### 4.2. Plasmids and Transfection

The plasmids encoding wild-type EBOV (pCAGGS-NP, -VP24, and -VP35) proteins and marker-fused protein-expressing plasmids for EBOV pCAGGS-VP30-GFP, pCAGGS-VP24-TagRFP, and MARV pCAGGS-VP30-GFP were expressed as described previously [15,27,43,44]. For nucleocapsid-like structure (NCLS) experiments, Huh7 cells were transfected with pCAGGS-NP, VP24, VP35, and VP30-GFP (200 ng each) using TransIT-LT1 (Mirus, Madison, WI, USA) according to the manufacturer’s instructions. At 24 h post-transfection, NCLS movement was recorded by detecting VP30-GFP. TagRFP-actin was used for actin visualization [27]. TagRFP-actin was transfected with NCLS plasmids to visualize the interaction between NCLS and actin.

### 4.3. Infection and Transfection of Cells in BSL-4

Huh7 cells seeded in 8-well slides were infected with either EBOV or MARV at a multiplicity of infection (MOI) of 1 in 250 µL DMEM/PS/Q without FCS. After 1h, the inoculum was replaced with 250 µL of the same medium. Afterwards, 500 ng of EBOV pCAGGS-VP30-GFP + pCAGGS-VP24-TagRFP or MARV pCAGGS-VP30-GFP were transfected for nucleocapsid tracking. One hour after transfection, the medium was replaced with a fresh medium.

### 4.4. Live-Cell Microscopy

Both NCLS and live virus movement were observed using live-cell time-lapse experiments. The NCLS experiments were recorded in BSL-2 using a Nikon ECLIPSE TE2000-E microscope (Düsseldorf, Germany) with a 63× oil objective lens or Keyence BZ-X800 microscope with (Tokyo, Japan) a 100× oil objective lens. Live virus-infected cells were recorded in BSL-4 using a Leica DMI6000B (Wetzlar, Germany) with a 63× oil objective lens. Image and video processing were performed with Nikon NIS Element 4.1 software and Keyence BZ-X series 1.3 software or Leica Application Suite X 3.10.0 Software, respectively, and edited with ImageJ. All experiments were performed at least in triplicate.

## Data Availability

The film is available as part of the Supplementary Materials.

## Supplementary Materials

The following supporting information can be downloaded at https://doi.org/10.5281/zenodo.16690788.

## Abbreviations

The following abbreviations are used in this manuscript:

EBOV	Ebola virus
MARV	Marburg virus
GP	Glycoprotein
RNP	Ribonucleoprotein
NP	Nucleoprotein
L	L polymerase
VP35	Polymerase cofactor
VP30	Transcriptional activator
VP24	Viral protein 24
VP40	Matrix protein
Arp2/3	Actin related protein 2/3
TNT	Tunneling nanotube
NCLS	Nucleocapsid-like structure
GFP	Green fluorescent protein
TagRFP	Red fluorescent protein
